# Immune Activation Response in Chronic HIV-Infected Patients: Influence of Hepatitis C Virus Coinfection

**DOI:** 10.1371/journal.pone.0119568

**Published:** 2015-03-16

**Authors:** Mercedes Márquez, Paula Romero-Cores, Monserrat Montes-Oca, Andrés Martín-Aspas, María-José Soto-Cárdenas, Francisca Guerrero, Clotilde Fernández-Gutiérrez, José-Antonio Girón-González

**Affiliations:** 1 Servicio de Medicina Interna/Enfermedades Infecciosas, Hospital Universitario Puerta del Mar/Universidad de Cádiz, Cádiz, Spain; 2 Servicio de Microbiología, Hospital Universitario Puerta del Mar/Universidad de Cádiz, Cádiz, Spain; Karolinska Institutet, SWEDEN

## Abstract

**Objectives:**

We have analyzed the parameters (bacterial translocation, immune activation and regulation, presence of HCV coinfection) which could be implicated in an inappropriate immune response from individuals with chronic HIV infection. The influence of them on the evolution of CD4+ T cell count has been investigated.

**Patients and methods:**

Seventy HIV-infected patients [monoinfected by HIV (n = 20), HCV-coinfected (with (n = 25) and without (n = 25) liver cirrhosis)] and 25 healthy controls were included. Median duration of HIV infection was 20 years. HIV- and HCV-related parameters, as well as markers relative to bacterial translocation, monocyte and lymphocyte activation and regulation were considered as independent variables. Dependent variables were the increase of CD4+ T cell count during the follow-up (12 months).

**Results:**

Increased values of bacterial translocation, measured by lipopolysaccharide-binding protein, monocyte and lymphocyte activation markers and T regulatory lymphocytes were detected in HIV-monoinfected and HIV/HCV coinfected patients. Serum sCD14 and IL-6 were increased in HIV/HCV-coinfected patients with liver cirrhosis in comparison with those with chronic hepatitis or HIV-monoinfected individuals. Time with undetectable HIV load was not related with these parameters. The presence of cirrhosis was negatively associated with a CD4+ T cell count increase.

**Conclusion:**

In patients with a chronic HIV infection, a persistent increase of lipopolysaccharide-binding protein and monocyte and lymphocyte modifications are present. HCV-related cirrhosis is associated with more elevated serum concentrations of monocyte-derived markers. Cirrhosis influences the continued immune reconstitution of these patients.

## Introduction

Antiretroviral treatment (ART) has been implicated in the transformation of human immunodeficiency virus (HIV) infection in a chronic entity. The undetectability of plasma HIV load in HIV-infected patients on ART is followed-up by immunological reconstitution, typically measured by circulating CD4+ cell counts [1,2]. However, a percentage of these patients, oscillating between 7% and 47%, shows an insufficient increase of CD4+ T cells even if plasma HIV load remains undetectable during prolonged periods [3,4]. Causes of discordant immune response are multiple. Among them it has been considered: 1) The persistence of immune activation [5,6]. Immune activation has been related with the following features: a) persistence of HIV replication in lymphatic nodes, although peripheral blood HIV replication was controlled [7]; b) bacterial translocation, related with the damage of intestinal barrier by the HIV [7], and c) existence of other coinfections, mainly cytomegalovirus infection [8]. 2) Immune activation induces an increase of regulatory T lymphocytes (Treg) (CD4+CD25highCD127lowFoxP3+) [9]. Cell contact and Treg-dependent secretion of transforming growth factor beta 1 (TGF-β1) modulate the lymphocyte proliferation, and it might contribute to the immune discordant response [10]. 3) Fibrosis of lymphatic nodes, related, among others, with the secretion of TGF-β1, interferes with the lymphocyte proliferation in these organs [11]. 4) Absence of an adequate tymopoiesis caused by either alteration of lymphocyte precursors or aging [12].

Hepatitis C virus infection is frequently detected in HIV-infected patients when the transmission risk is related with the parenteral use of drugs or hemoderivatives administration. HCV coinfection has been scarcely studied as a pathogenic factor of the immune discordant response [13,14], even though HCV might be implicated in it because of its effect on immune activation [15], the increase of Treg-dependent TGF-β1 secretion [16], or because of the bacterial translocation associated with portal hypertension, a situation typical of advanced phases of hepatopathy [17].

The absence of an adequate immune response is more complex in patients after several years of ART. Among individuals with virological suppression, CD4+ cell counts continue to increase throughout 5 years, although the rate of CD4 count increase diminishes with the prolongation of time since starting ART [18]. Cohort studies have observed that CD4 cell counts may reach a plateau after the first years of ART and that patients who started ART with CD4+ cell counts < 350 cells/mm3 were less likely to achieve normal levels [19,20,21]. Possible causes of this altered immune reconstitution are not clarified.

In this work, bacterial translocation and monocyte and lymphocyte activation and regulatory markers were analysed in a series of HIV-infected patients with a prolonged period of infection. The influence on these parameters of the time with ART-induced undetectable HIV load and the presence of HCV coinfection and its consequences was studied. In a different analysis, the influence of bacterial translocation and immune markers on the evolution of CD4+ T cell count after a 12-month period has also been prospectively investigated.

## Patients and Methods

### Patients and controls

Seventy HIV-infected patients were prospectively included. All subjects were consecutively recruited from a prospectively collected cohort of HIV-infected patients treated at the HIV outpatients’ clinics of a university hospital. Patients were distributed in two groups: those monoinfected by HIV (n = 20) and those with HCV-coinfection, grouped in those with (n = 25) and those without (n = 25) liver cirrhosis.

Inclusion criteria were: 1) Infection by HIV. 2) To be on ART. 3) Achievement and persistence of undetectable HIV load during at least 12 months. Exclusion criteria were as follows: (1) Active opportunistic or concomitant infections. (2) Neoplasias, including liver cancer at inclusion. (3) Evidence of active infection by hepatitis B virus (HbsAg negative), alcoholic hepatitis, or metabolic or autoimmune liver disease. Significant alcohol ingestion (higher than 50 g/day during at least 5 years) was also a criterion of exclusion. (4) Decompensation of liver cirrhosis. (5) Treatments which could have modified the determination of cytokines (pentoxyfilline, steroidal, or nonsteroidal anti-inflammatory or immunosuppressive drugs). (6) Treatment against chronic HCV infection. (7) Red blood cell or plasma transfusion in the month before inclusion in the study.

As a control group, we studied a sample of healthy subjects (n = 25) recruited from voluntary hospital workers, whose age and gender were comparable with those of the patients.

### Definitions

Patients were followed-up in our hospital with clinical and analytical revision every three months. Positive serum antibodies against HIV were required for the diagnosis of HIV infection. Patients were classified according the 1993 Centers for Disease Control and Prevention classification of HIV infection. Spanish Group for AIDS Study guidelines (www.gesida.es) was used to indicate the antiretroviral treatment (ART). Plasma HIV RNA load lower than 50 copies/ml was considered as undetectable HIV load.

Positive serum antibodies against HCV and persistent (more than 6 months) HCV RNA were required for the diagnosis of chronic HCV infection. Diagnosis of chronic hepatitis or cirrhosis was established according to histological criteria when liver biopsy was performed [22], or by transient elastography, performed according to a standardized technique by one trained operator (JAGG) (according to data validated in HIV-HCV coinfected patients using liver biopsy as reference, patients with a liver stiffness > 14,6 kPa were classified as individuals with cirrhosis) [23].

Duration of HIV infection was estimated using an interviewer-assisted questionnaire assessing risk factors for HIV infection. The earliest exposure was designated as the time of acquisition [24].

### Phenotypic studies

**Fig 1:**
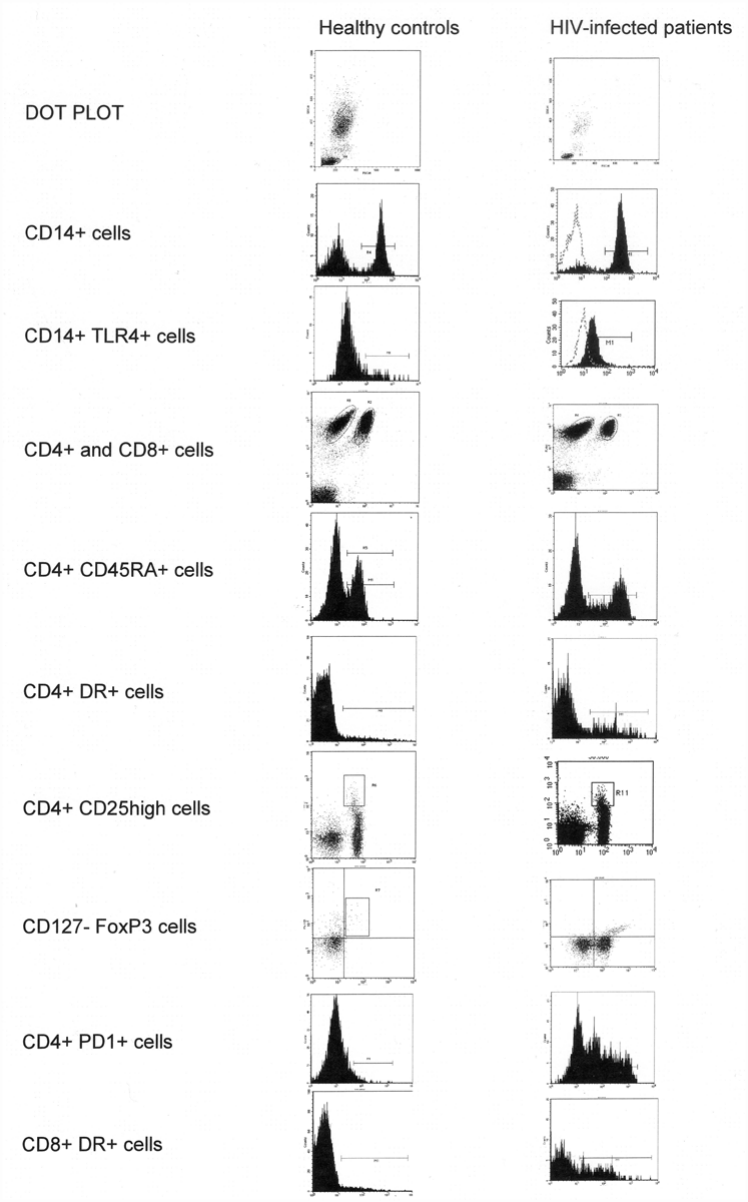
FACS gating strategy and plots for all the cell subsets analysed in a representative healthy control and a HIV-infected patient.

Blood samples, collected in pyrogen-free heparinized tubes (Biofreeze, Costar, EEUU), were taken at 8 am to minimize the influence of circadian rhythms. Peripheral blood mononuclear cells (PBMC) were obtained from heparinized venous blood by Ficoll-Hypaque (Lymphprep Nyegaard, Oslo, Norway) density gradient centrifugation. PBMC were incubated with combinations of fluorescein-CD127 (FITC), (Biolegend, San Diego CA, USA) phycoerythrin-CD25 (PE) (Becton-Dickinson, San Jose, CA, USA) and peridinin chlorophyll protein CD4 (PerCP) (Becton-Dickinson), fluorescein-CD45RA (FITC) (Becton-Dickinson), fluorescein-CD279 [–anti-PD1-] (FITC) (Becton-Dickinson), fluorescein-CD284 [–anti-TLR4-] (FITC) (Imgenex, San Diego CA, USA), and peridin chlorophyll protein CD14 (PerCP) (Becton-Dickinson) labelled monoclonal antibodies. The anti-human FOXP3 APC (eBioscience, San Diego CA, USA) was used for intracellular T cell immunophenotyping. Stained cells were washed, acquired, and analyzed by two-colour flow cytometry in a FACScalibur cytometer using Cell Quest and Paint-A-Gate software (Becton-Dickinson). FACS gating strategy and plots for all the cell subsets analysed are shown in Fig. 1.

### Concentration of LBP and of pro- and anti-inflammatory molecules

Serum LBP was measured by immunometric sandwich assay (Immulite LBP; DPC, Los Angeles, CA). Serum concentrations of soluble CD14 (sCD14) and IL-6 were analysed using Milliplex MAP kit High Sensitivity Human Cytokine (Millipore, Billerica, MA, USA). Serum TGF-β1 levels were detected by Quantikine Human Immunoassay (R&D, Minneapolis, MN, USA).

### Statistical analysis

Descriptive data were expressed as the median (25–75 interquartile range—IQR-) or as an absolute number (percentage). Qualitative variables were compared by the chi-square test or Fisher’s exact test when necessary. Quantitative variables were compared using the Mann-Whitney U test or ANOVA when necessary. The Spearman’s correlation test analysed the association between quantitative variables.

To analyse the influence of the time with plasma HIV undetectability on the bacterial translocation and immune response detected at the inclusion in the study, patients were grouped in function of the time (12–36, 36–60, > 60 months) with a continued plasma HIV load < 50 copies/ml. The influence on them of HCV coinfection and the presence or absence of liver cirrhosis was also investigated.

In a different analysis, the parameters influencing the increase of CD4+ T cells during a prospective 12-month period were considered. In function of the median of the increase of CD4+ T cells after 12 months of follow-up after inclusion, patients were classified in two groups: those with an adequate CD4+ T cell increase and those with an inadequate response. Demographic, HIV-related (CD4+ T cells at diagnosis and at inclusion in the study, protease inhibitor—based ART, previous time with undetectable HIV load) and HCV-related (percentage of patients with coinfection by HCV and of patients with liver cirrhosis) parameters, as well as parameters relative to bacterial translocation, monocyte and lymphocyte activation, and markers of Treg lymphocytes were considered as possible independent variables in this analysis. After a bivariant study, these variables with a significance lower than 0,1 were introduced in a linear regression study.

A *p* value <0.05 was considered significant. The statistical analysis was carried out using the SPSS 15.0 statistical software package (SPSS Inc., Chicago, IL, USA).

### Ethical aspects

This study was performed according to the Helsinki Declaration. The project was approved by the University Hospital Puerta del Mar (Cádiz, Spain) ethical research committee. Written informed consent was obtained from each participant.

## Results

Characteristics of the patients are shown in Table 1. Patients with chronic hepatitis secondary to HIV/HVC coinfection had higher CD4+ T cell count at inclusion than those with HIV monoinfection and those with liver cirrhosis. Due to these differences, data about lymphocyte subpopulations will be expressed as percentages.

**Table 1:** General characteristics of the HIV-infected patients (n = 70).

	Global (n = 70)	HIV-monoinfected patients (n = 20)	HIV/HVC coinfected patients		
			Global (n = 50)	Chronic hepatitis (n = 25)	Liver cirrhosis (n = 25)
**General characteristics**					
Age (years)	47 (42–49)	46 (39–49)	47 (43–50)	47 (43–51)	47 (43–49)
Sex male (n, %)	59 (84)	16 (80)	43 (86)	20 (80)	23 (92)
Previous parenteral drug use (n, %)	44 (63)	12 (60)	32 (64)	14 (56)	18 (72)
Evolution of the infection (years)	22 (19–26)	22 (19–24)	22 (20–27)	22 (20–26)	22 (19–27)
**HIV-related characteristics**					
CD4+ T cell/mm3 at diagnosis	150 (87–267)	182 (107–416)	146 (65–253)	160 (73–258)	145 (64–234)
CD4+ T cell/mm3 at inclusion	342 (252–535)	305 (193–473)	359 (277–540)	519 (321–661) *	339 (229–380) *
CD4+ T cell increase from diagnosis until inclusion	181 (57–295)	85 (-53; +183)	202 (111–334)	288 (177–399)	172 (55–221)
CDC C stage (n, %)	36 (51)	12 (60)	24 (48)	14 (56)	10 (40)
Months with undetectable HIV load	42 (17–72)	49 (16–102)	39 (18–62)	24 (16–60)	48 (18–55)
Protease inhibitor—based HAART (n, %)	38 (54)	11 (55)	27 (54)	13 (52)	14 (56)
**HCV-related characteristics**					
Liver stiffness (kPa)			15 (10–27)	10 (9–12) **	27 (17–33) **
HCV genotype 1 or 4 (n, %)			32 (64)	18 (72)	14 (56)
HCV viral load (x 1000) at inclusion			2574 (401–4640)	3130 (643–4404)	2514 (572–4948)

Quantitative variables are expressed as median (interquartile range).

* p = 0,003 HIV/HCV coinfected patients with cirrhosis vs HIV/HCV coinfected patients with chronic hepatitis.

** p < 0,001 HIV/HCV coinfected patients with cirrhosis vs HIV/HCV coinfected patients with chronic hepatitis.

### Bacterial translocation and monocyte and lymphocyte parameters and relation with HCV coinfection

Serum levels of LBP were significantly elevated in HIV-infected patients with reference to healthy controls (p<0,001). Concentrations of LBP were similar in HIV-infected patients with or without HCV coinfection (with or without liver cirrhosis) (p>0,05) (Table 2).

**Table 2:** Bacterial translocation, monocyte and lymphocyte markers and Treg-derived parameters in healthy controls and HIV-infected patients with or without coinfection by HCV.

	Healthy controls (n = 25)	HIV- infected patients				
		Global(n = 70)	HIV-mono-infected (n = 20)	HIV-HCV coinfected patients		
				Global (n = 50)	Chronic hepatitis (n = 25)	Liver cirrhosis (n = 25)
**Bacterial translocation**						
LBP (ng/ml)	4 (3–4)	9 (6–14) *	8 (5–14)	10 (7–14)	11 (8–12)	9 (6–18)
**Monocyte parameters**						
CD14+TLR4+ cells (% of total CD14+ cells)	19 (11–28)	83 (68–88) *	83 (67–87)	82 (68–88)	84 (78–91)	79 (59–85)
Serum soluble CD14 (x 100) (ng/ml)	10 (5–12)	19 (14–27) *	15 (8–24)	21 (16–28)	19 (16–25)	26 (16–30) **
Serum IL-6 (pg/ml)	2 (1–6)	6 (2–15)	6 (2–13)	9 (2–15)	6 (5–8)	14 (8–18) **
**Lymphocyte parameters**						
CD4+ T cells (% of total T cells)	58 (48–62)	27 (19–34) *	22 (19–36)	29 (18–34)	26 (24–33)	29 (18–36)
CD4+DR+ cells (% of total CD4+T cells)	12 (9–14)	18 (14–25) *	21 (16–66)	17 (14–23)	14 (12–21)	18 (15–25)
CD4+CD45RA+ cells (% of total CD4+T cells)	49 (36–62)	37 (21–45) *	31 (19–40)	39 (22–45)	42 (21–48)	38 (22–45)
CD4+PD1+ cells (% of total CD4+T cells)	5 (2–10)	21 (8–55) *	26 (12–81)	17 (8–51)	16 (5–70)	17 (8–51)
CD8+ T cells (% of total T cells)	24 (20–27)	35 (30–45) *	44 (19–57)	36 (31–44)	39 (31–45)	35 (31–42)
CD8+DR+ cells (% of total CD8+T cells)	22 (17–26)	38 (39–44) *	38 (35–81)	39 (27–44)	35 (30–43)	42 (20–44)
CD4 / CD8 ratio	1,7 (1,1–2,2)	0,8 (0,5–1,0) *	0,5 (0,4–2,1)	0,9 (0,5–1,0)	0,8 (0,5–1,0)	0,8 (0,5–1,1)
**T regulatory cells parameters**						
CD4+CD25highCD127lowFoxP3+ (% of total CD4+T cells)	1 (0–2)	7 (3–11) *	8 (3–10)	7 (3–12)	5 (4–16)	8 (3–14)
Serum TGF-β1 (pg/ml)	38 (19–49)	72 (52–97) *	77 (58–121)	72 (49–97)	83 (58–115)	70 (45–89)

Variables are expressed as median (interquartile range).

* p < 0,05 comparing healthy controls with HIV-infected patients, global.

** p < 0,05 comparing patients with chronic hepatitis and cirrhosis.

Parameters indicative of monocyte activation (percentage of CD14+TLR4+ and serum sCD14 and IL-6 levels) were significantly increased in the overall group of HIV-infected patients (p<0,001) (Table 2). There was no significant difference in these parameters when patients with HIV monoinfection were compared with those with HIV/HCV coinfection with chronic hepatitis. Patients with cirrhosis showed significantly higher concentrations of serum sCD14 (p = 0,024) and IL-6 (p = 0,036) than those with chronic hepatitis.

The correlations between serum LBP levels and monocyte parameters (CD14+TLR4+ percentage and sCD14 and IL-6) were not significant (p>0,05 in each case).

At inclusion, HIV-infected patients showed a lower percentage of CD4+ T cells (p<0,001) and a higher percentage of CD8+ T cells (p = 0,005) than healthy controls, with a decreased CD4/CD8 ratio (p = 0,001). Percentages of activated CD4 (CD4+DR+) and CD8 (CD8+DR+) T lymphocytes were significantly higher in HIV-infected patients than in healthy controls (p = 0,041 and p = 0,048, respectively). HIV-infected patients showed lower percentages of naïve T CD4+ lymphocytes (CD4+CD45RA+) than healthy controls (p = 0,040). Finally, CD4+ T cells expressing the death receptor PD-1 were significantly increased in HIV-infected individuals with reference to healthy controls (p = 0,017). There was no significant difference in these parameters when patients with HIV monoinfection were compared with those with HIV/HCV coinfection. Likewise, there was no significant difference in these parameters when patients with chronic hepatitis were compared with those with liver cirrhosis (Table 2).

A significant correlation was established between serum LBP levels and the percentage of CD4+DR+ (r = 0,842, p = 0,004) and CD8+DR+ (r = 0,561, p = 0,016). A negative correlation was detected between percentages of activated CD4+ cells (CD4+DR+) and naïve CD4+ cells (CD4+CD45RA+) (r = -0,467, p = 0,025). No significant correlation was detected between CD4+DR+ percentage and monocyte (CD14+TLR4+, serum levels of sCD14 and IL-6) parameters (p>0,05 in each case).

The percentage of Treg lymphocytes and serum concentrations of TGF-β1 were significantly elevated in HIV-infected patients with reference to healthy controls (p<0,001 in each case). Treg percentage and serum TGF-β1 levels were similar in HIV/HCV coinfected patients (without differences between those with chronic hepatitis and those with liver cirrhosis) and HIV-monoinfected individuals (p>0,05, in each case). A significant correlation was demonstrated between the percentage of Treg lymphocytes and TGF-β1 (r = 0,921, p = 0,001) in these patients.

No significant correlation was established between CD4+DR+ and Treg percentages (p>0,05). Serum levels of TGF-β1 and IL-6 (r = -0,246, p = 0,043) were significantly correlated.

### Relation between the time with undetectable HIV load and markers of bacterial translocation and lymphocyte or monocyte activation and regulation

**Fig 2:**
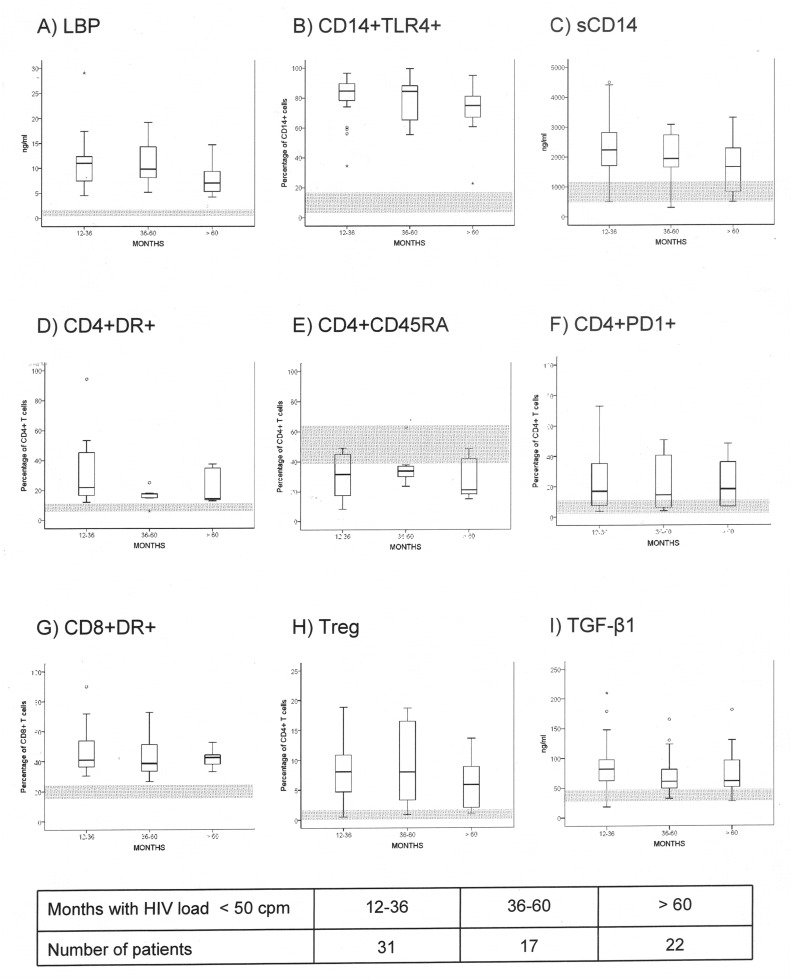
Markers of bacterial traslocation (A) (serum levels of lipopolysaccharide binding protein—LBP-, ng/ml); monocyte derived parameters [toll-like receptor 4 expression on monocyte (B) (CD14+TLR4+, percentage of blood CD14+ cells) and soluble CD14 receptor (C) (sCD14, ng/ml)], T CD4+-related populations [activated CD4+ T cells (D) (CD4+DR+, percentage of blood T CD4+ cells), naïve T CD4+ cells (E) (CD4+CD45RA+, percentage of blood T CD4+ cells), CD4+ T cells expressing the dead receptor PD1 (F) (CD4+PD1+, percentage of blood T CD4+ cells)], activated T CD8+ cells (G) (CD8+DR+, percentage of blood T CD8+ cells), and regulatory T cells [Treg, CD4+CD25highCD127lowFoxP3+ T cells (H) (percentage of blood T CD4+ cells) and serum concentration of transforming growth factor beta 1 (I) (TGF-β1, ng/ml)] in patients with HIV load undetectable for 12–36, 36–60 or > 60 months. Grey areas represent the normal values (range) of each parameter in healthy controls.

Patients included in the study had shown continuous undetectable HIV load during a median of 44 months (range: 13–132 months) and 21 patients (30%) of them showed a normal CD4+ T cell count (≤ 500/mm3). Even in patients with more than 60 months with undetectable HIV load, LBP, monocyte and lymphocyte activation markers, and Treg percentage and TGF-β1 persisted elevated in comparison with values of the healthy controls (Fig. 2).

### Implications of bacterial translocation and immune parameters on the magnitude of increase in CD4+ T cell count during the follow-up

Patients were followed-up during 12 months after the beginning of the study. The median increase of CD4+ T cell lymphocytes was 10 (IQR-74; +122)/mm3. Patients were distributed in two groups in function of that their increase of CD4+ T cells were higher or lower than 10 cells/mm3 (the value of the median). Patients with an increase of CD4+ T cells higher than the median showed a CD4+ T cell gain of 110/mm3 (IQR +40; +156), whereas those with an increase of CD4+ T cells lower than the median showed a CD4+ T cell decrease of-90/mm3 (IQR, -193; -31). The absolute CD4+ T cell count after 12 months of follow-up was 331 (IQR, 169; 469) cells/mm3 in patients with an increase lower than the median and 398 (IQR, 283; 671) cells/mm3 in those with an increase higher than the median (p<0,001).

Differential characteristics are shown in the Table 3. Only a higher CD4+ cell increase from HIV diagnosis until the beginning of the study and a higher CD4+ cell count at inclusion were significantly associated with a lower increase of CD4+ cells during the follow-up. The presence of cirrhosis approaches significance. Values of bacterial translocation and immune parameters were similar in patients with a higher or lower increase of CD4+ cells. When only HIV/HCV coinfected patients were considered, correlation among CD4+ cell count increase and liver stiffness was not significant (r = -0,175, p = 0,322). No significant correlation was established between time with undetectable HIV load and CD4+ cell increase (r = 0,113, p = 0,365).

**Table 3:** Lymphocyte and monocyte activation markers, T reg lymphocytes and bacterial translocation markers at inclusion in patients with an increase or decrease of CD4+ T cells/mm3 during the follow-up with reference to the median.

	Patients with an increase of CD4 T cells lower than 10 / mm3 (n = 37)	Patients with an increase of CD4 T cells higher than 10 / mm3 (n = 33)	P
**General characteristics**			
Age (years)	47 (43–49)	45 (42–51)	0,533
Sex male (n, %)	32 (87)	27 (82)	0,745
**HIV-related characteristics**			
Time of evolution of HIV infection (years)	22 (19–26)	22 (19–27)	0,892
CD4+ T cell/mm3 count at diagnosis	188 (89–310)	146 (80–209)	0,380
CD4+ T cell/mm3 count at inclusion	381 (315–558)	300 (197–395)	0,008
Time with undetectable HIV load (months)	39 (14–62)	48 (14–72)	0,504
Protease inhibitor based HAART (n, %)	19 (52)	19 (58)	0,420
Increase of CD4+ T cell/mm3 count from nadir until inclusion	221 (57–344)	168 (50–283)	0,047
Increase of CD4+ T cell/mm3 count during follow-up	-90 (-193; -31)	110 (+40; +156)	0,001
**HCV-related parameters**			
Patients with HCV coinfection (% of total of patients)	29 (78)	21 (64)	0,196
Patients with liver cirrhosis (% of total of patients)	17 (46)	8 (24)	0,081
**Bacterial translocation**			
LBP (ng/ml)	8 (6–12)	12 (7–14)	0,760
**Monocyte and lymphocyte derived parameters**			
CD14+TLR4+ cells (% of total CD14+ cells)	84 (75–90)	78 (63–87)	0,188
Serum soluble CD14 (x 100) (ng/ml)	21 (16–27)	19 (9–26)	0,318
Serum IL-6 (pg/ml)	9 (3–15)	5 (1–13)	0,270
CD4+ T cells (% of total lymphocytes)	30 (18–37)	25 (20–30)	0,286
CD4+DR+ cells (% of total CD4+T cells)	17 (14–23)	20 (15–35)	0,870
CD4+CD45RA+ cells (% of total CD4+T cells)	32 (25–47)	28 (18–41)	0,215
CD4+PD1+ cells (% of total CD4+T cells)	26 (7–42)	34 (10–76)	0,299
CD8+ T cells (% of total T cells)	34 (26–43)	42 (33–52)	0,094
CD8+DR+ cells (% of total CD8+T cells)	32 (19–43)	43 (37–55)	0,174
CD4/CD8 ratio	0,8 (0,5–1,4)	0,6 (0,4–1,0)	0,278
**T regulatory cells number and function**			
CD4+CD25highCD127lowFoxP3+ (% of total CD4+T cells)	7 (4–14)	7 (2–11)	0,580
Serum TGF- β1 (pg/ml)	69 (49–96)	75 (58–115)	0,265

Quantitative variables are expressed as median (interquartile range).

**Fig 3:**
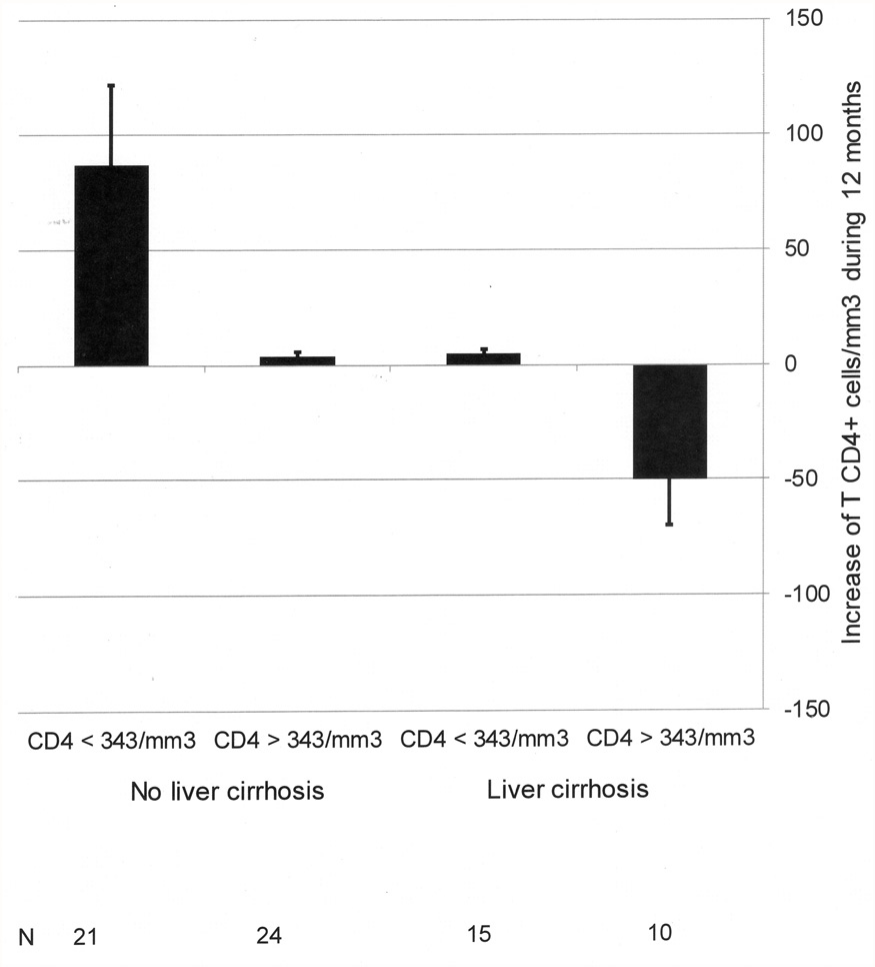
Increase in CD4+ T cell count during a follow-up of 12 months in HIV-infected patients with undetectable HIV viral load. Patients have been classified in function of: 1) CD4+ T cell count at the beginning, and 2) presence or absence of liver cirrhosis.

Logistic regression analysis of those factors associated with the increase of CD4+ cell count showed that a CD4+ cell count lower than the median (343/mm3) at the beginning of the study (Exp(B) 5,354, 95% confidence interval 1,681–17,052, p = 0,005) and the absence of cirrhosis (Exp(B) 4,648, 95% confidence interval 1,437–15,034, p = 0,010) were positively associated with the increase of CD4+ cell during the follow-up. The increase of CD4+ cell count in function of the presence or absence of these factors is shown in Fig. 3.

## Discussion

In a sample of chronically HIV-infected patients, we have studied their alterations in intestinal permeability, the monocyte and lymphocyte activation, and the regulatory T cells number.

Both HIV-monoinfected and HIV/HCV coinfected patients showed a state of bacterial translocation, as revealed by the increased LBP levels. LBP was selected as a measure of bacterial translocation because it is correlated with LPS concentration [25] but is more reliable than LPS due to its longer half-life [26]. Serum LBP levels in patients with or without HCV-coinfection were similar: it must be considered that decompensated cirrhosis, a situation in which bacterial translocation is characteristic [27], was an exclusion criterion.

Chronic monocyte and lymphocyte activation has been previously described in HIV-infected patients [28,29]. Markers of monocyte activation (sCD14 and IL-6) have been considered as powerful predictive parameters of evolution of HIV-infection, due to both AIDS- and non-AIDS related entities [30,31], and they have been proposed in risk stratification strategies [32].

As it must be expected in a situation of chronic antigenemia [33,34,35], an increase of regulatory T lymphocytes (Treg) and serum TGF-β1 concentration was observed. High frequencies of Tregs can down-regulate immune activation but also benefit HIV-specific responses, thereby favouring persistence of HIV infection [36,37]. Interestingly, our results demonstrate that serum concentration of TGF-β1, the main molecule secreted by Treg, significantly correlate with those of IL-6, suggesting that monocyte activation might be a powerful stimulating factor of secretion of TGF-β1.

The proportion of naïve T cells was decreased and the percentage of cells prone to apoptosis, as revealed by the expression of the programmed death receptor PD-1 on CD4+ cells, was increased in HIV-infected patients, with independence of the evolution of CD4+ cell number. Recent in vitro and in vivo studies, including those performed in HIV-infected individuals, have shown the importance of the PD-1 interaction with its ligand PD-L1 in the pathogenesis of decreased T cell proliferation and increased apoptosis [38].

Our work has analysed if these abnormalities in intestinal permeability and in monocyte and lymphocyte functions are related with the time of undetectable HIV load as well as with the presence of HCV coinfection. Antiretroviral treatment decreases but does not normalize bacterial translocation markers when it is initiated in patients with recent HIV infection [28]. Interestingly, in patients such as those in our series with a more chronic HIV infection, LBP values kept on having increased and similar to baseline levels even in patients with more than 5 years of continuous undetectable HIV load.

A rapid reduction in both activated CD4+ and CD8+ cell populations coincides with the rapid first phase of immune reconstitution and control of HIV viremia, supporting the hypothesis that ART reduces inflammation and subsequent redistribution of CD4+ and CD8+ cells [7,8,28]. However, in patients with chronic HIV infection with a time of infection of more than 20 years, such as those studied in the present work, the immune activation state is maintained.

The influence of HCV coinfection and liver cirrhosis was also considered. Data of our work have demonstrated that both sCD14 and IL-6 are even more increased in those HIV-infected patients with HCV-related cirrhosis. Previous results of our group had detected the elevation of these markers in patients with decompensated liver cirrhosis, having been attributed to the situation of bacterial translocation associated with the portal hypertension [17,34]. But in the present work, the elevation of sCD14 and IL-6, but not of CD14+TLR4+ percentage or lymphocyte activation, was also evident in patients with HCV-related compensated cirrhosis. There was no significant difference between patients with HIV monoinfection and those with chronic hepatitis. Moreover, HCV load was similar in HIV/HCV coinfected patients with chronic hepatitis and liver cirrhosis (data not shown), discarding the possibility of an effect directly attributed to the hepatitis virus on monocyte activation. A possible explanation for this finding has been provided by the experimental demonstration of a monocyte activation state directly associated with the liver cirrhosis: effectively, monocyte activation is present in hepatic lymph nodes, and serum IL-6 is increased in cirrhotic rats previously to the development of portal hypertension [39]. Thus, both HCV-related liver cirrhosis and HIV-induced intestinal barrier lesion could be operative and justify the increased values of sCD14 and IL-6 in HIV/HCV cirrhotic patients. This double influence on monocyte activation could explain the absence of significant correlation among sCD14 or IL-6 and LBP, observed in this and in a previous study [40]. Taking into account the predictive value of sCD14 and IL-6, this can also explain the increased mortality of HIV/HCV coinfected patients detected by some authors [41,42] and the beneficial impact of HCV eradication in cirrhotic patients on survival [43].

Finally, the influence of the several HIV- and HCV-related parameters, as well as the alterations in immune responses, on the evolution of CD4+ cell count has been studied. Among individuals with virological suppression, CD4+ cell counts continue to increase throughout 5 years [18], although patients who started ART with CD4+ cell counts < 350 cells/mm3 were less likely to achieve normal levels [19,20,21,44]. Effectively, a notable percentage (40%) of the patients did not increase the CD4+ cell count after 5 years of undetectable HIV load [45]. These findings led to the conclusion that not all patients might eventually respond to ART by achieving a CD4 count in the normal range. As it has been discussed, patients in our series showed several characteristics previously associated with a poor immune reconstitution: immune activation, increased expression of PD-1, decreased proportion of naïve CD4+ T cells, and elevated percentages of Treg [5,10,11]. These parameters were similar in groups in which occurred or not occurred the increase of CD4+ T cell counts, implicating that this alteration of the innate and adaptive immune systems is not a primary causative factor of the absence of CD4+ cell increase in this population with a prolonged period of infection.

Furthermore, HCV infection was considered as a factor implicated in the presence or absence of increase of CD4+ cell count. HCV coinfection has been previously analysed in the pathogenesis of discordant immune response, discarding its independent contribution to it [6,18]. However, the importance of the cirrhosis, and not of every histological type of HCV-related liver disease, has not been previously studied. The presence of cirrhosis was negatively associated with an increase of CD4+ cell count. This is the first time in which the influence of cirrhosis has been considered a parameter implicated in the altered immune recuperation of HIV-infected individuals. It is possible that the increase of serum levels of sCD14 and IL-6, particularly elevated in cirrhotic patients, compromises the immune reconstitution, although they were not independent factors associated with it.

In conclusion, this work has demonstrated that in patients with a chronic HIV infection, a persistent increase in intestinal permeability and immune modifications are present. HCV-related cirrhosis is associated with more elevated serum concentrations of monocyte-derived markers (sCD14 and IL-6). Furthermore, cirrhosis influences the continued immune reconstitution of these patients.
